# iTRAQ protein profile analysis of neuroblastoma (NA) cells infected with the rabies viruses rHep-Flury and Hep-dG

**DOI:** 10.3389/fmicb.2015.00691

**Published:** 2015-07-07

**Authors:** Youtian Yang, Wenjun Liu, Guangrong Yan, Yongwen Luo, Jing Zhao, Xianfeng Yang, Mingzhu Mei, Xiaowei Wu, Xiaofeng Guo

**Affiliations:** ^1^College of Veterinary Medicine, South China Agricultural UniversityGuangzhou, China; ^2^Institute of Life and Health Engineering and National Engineering and Research Center for Genetic Medicine, Jinan UniversityGuangzhou, China

**Keywords:** rabies virus, glycoprotein, NA, iTRAQ, interferon

## Abstract

The rabies virus (RABV) glycoprotein (G) is the principal contributor to the pathogenicity and protective immunity of RABV. In a previous work, we reported that recombinant rabies virus Hep-dG, which was generated by reverse genetics to carry two copies of the G-gene, showed lower virulence than the parental virus rHep-Flury in suckling mice with a better immune protection effect. To better understand the mechanisms underlying rabies virus attenuation and the role of glycoprotein G, isobaric tags for relative and absolute quantitation (iTRAQ) was performed to identify and quantify distinct proteins. 10 and 111 differentially expressed proteins were obtained in rHep-Flury and Hep-dG infection groups, respectively. Selected data were validated by western blot and qRT-PCR. Bioinformatics analysis of the distinct protein suggested that glycoprotein over-expression in the attenuated RABV strain can induce activation of the interferon signaling. Furthermore, it may promote the antiviral response, MHC-I mediated antigen-specific T cell immune response, apoptosis and autophagy in an IFN-dependent manner. These findings might not only improve the understanding of the dynamics of RABV and host interaction, but also help understand the mechanisms underlying innate and adaptive immunity during RABV infection.

## Introduction

Rabies is an important viral zoonosis that occurs in over 150 countries and territories, causing more than 70,000 human deaths annually (Wang et al., 2014a). It is caused by rabies virus (RABV), which is an enveloped and bullet-shaped virus belonging to the rhabdoviridae family and lyssavirus genus. The RABV's genome encodes five structure proteins, including the nucleoprotein (N), phosphoprotein (P), viral polymerase protein (L), transmembrane glycoprotein (G), and matrix protein (M) (Okumura and Harty, 2011). The rabies virus's genomic RNA combined with N, P, and L form the ribonucleoprotein (RNP), which plays an important role in viral transcription and replication (Albertini et al., 2011). The matrix protein plays an essential role in virus assembly and budding, and is responsible for recruiting RNPs to the cell membrane, their condensation into tight helical structures to facilitate the efficient virion egress (Mebatsion et al., 1996, 1999). In addition, it has also been shown to play a regulatory role in the balance between transcription and replication of RABV (Finke et al., 2003; Finke and Conzelmann, 2003).

RABV glycoprotein is the only surface protein and its ectodomain is the target for neutralizing antibodies (Benmansour et al., 1991; Flamand et al., 1993). It plays a pivotal role during the initial steps of the infectious cycle. On the one hand, it recognizes and binds to receptors such as nicotinic acetylcholine receptor (nAChR) (Lentz et al., 1982), neural cell adhesion molecule (NCAM) receptor (Thoulouze et al., 1998) and Low-affinity nerve-growth factor receptor (P75NTR) (Tuffereau et al., 1998). On the other hand, this glycoprotein mediates the fusion between viral envelope and cellular membrane in the acidic environment (Albertini et al., 2011). Accumulating evidence indicates that the RABV G protein plays an essential role in the viral innate and adaptive immunity (Li et al., 2012; Zhang et al., 2013a). Prior studies have identified G protein's antigenic site III as the major pathogenicity determinant (Dietzschold et al., 1983; Seif et al., 1985). However, a study demonstrated that the rabies virus strains CVS-B2c and CVS-N2c differ greatly in pathogenicity, but not at this specific site (Morimoto et al., 1999). Meanwhile, the less pathogenic CVS-B2c virus expresses higher levels of G protein than does CVS-N2c in infected neurons. Moreover, the high G protein expression level in attenuated RABV has been correlated with apoptosis induction and higher levels of neutralizing antibodies (Faber et al., 2002; Hosokawa-Muto et al., 2006; Xue et al., 2014).

Similar to the above findings, we reported that recombinant rabies virus Hep-dG, which was generated by reverse genetics to carry two copies of the G-gene, showed decreased virulence than the parental virus rHep-Flury in suckling mice, with a better immune protection effect (Liu et al., 2014). Nevertheless, the mechanisms underlying rabies virus attenuation remain unclear. Interestingly, Faber et al demonstrated an association between apoptosis induction and immunogenicity of viral infection (Faber et al., 2002). In addition, we found that over-expression of RABV G protein induces apoptosis in BHK-21 (Liu et al., 2014) and NA cells. Furthermore, lower virus titers were produced in Hep-dG infected NA cells compared with those infected with rHep-Flury virus particles; this was not the case for BHK-21 cells (Liu et al., 2014). Similarly, prior studies have shown that recombinant rabies virus rHep-CaIFNα1, with insertion of the canine IFNα1, demonstrated much lower virus titer than parental virus rHep-Flury in NA cells and slightly lower titer in BSR cells (Wang et al., 2014b). Since BHK-21 cells are deficient in RIG-I-mediated IFN induction (Habjan et al., 2008), over-expression of RABV G proteins may result in increased interferon levels, which in turn likely contribute to the immunogenicity of the attenuated RABV. This conjecture has been proposed by Faber et al. (2002), but needs further verification in order to explain how G protein interacts with host cells and triggers the innate and adaptive immune responses.

Proteomics has been widely used in the study of virus infection, and the rabies virus infection research is no exception. Most of these studies based on 2D—LC/MS identified the important pathogenic mechanism of the rabies virus *in vivo* or *in vitro* (Dhingra et al., 2007; Zandi et al., 2009; Thanomsridetchai et al., 2011; Zhang et al., 2013b). In the present study, a modern quantitative proteomics approach was used to compare the proteomic changes of mouse neuroblastoma (NA) cells infected with rabies viruses rHep-Flury and Hep-dG. iTRAQ is a relatively new and popular LC-based technique, which employs a 4/8-plex set of amine reactive isobaric tags to derivatize peptides at the N-terminus and lysine side chains, thereby labeling all peptides in a digest mixture (Wu et al., 2006). Using this method, many cellular and possibly viral proteins were identified and quantified. Bioinformatics analysis of these distinct proteins suggested that interferon signaling was strongly activated in the Hep-dG infection group at a very low MOI of 0.01, but not in rHep-Flury-infected cells. Moreover, many interferon stimulated proteins which up-regulated in Hep-dG-infected cells were involved in antiviral response, antigen processing and presentation, cell apoptosis and autophagy. Type I IFNs are considered antiviral cytokines (Isaacs and Lindenmann, 1957), and stimulate the adaptive immunity by induction of immune-modulatory genes, supporting activation of dendritic cells (DCs), stimulating macrophages, increasing major histocompatibility complex class-I expression, stimulating antibody secretion, and thereby supporting a Th1-biased immune response, thus integrating innate and adaptive immunity (Rieder and Conzelmann, 2011). Our findings demonstrate a close and important relationship between the attenuated RABV G protein expression and the innate and adaptive immunity during virus infection.

## Materials and methods

### Cells and viruses

Mouse neuroblastoma (NA) cells (purchased from Wuhan Institute of Biological Products, China) were maintained in RPMI 1640 medium (Gibco, USA) supplemented with 10% fetal calf serum (Bioind, Israel). Recombinant RABV rHep-Flury and Hep-dG (which encode two copies of the G-gene) were generated from RABV Hep-Flury strain's full-length cDNA (Liu et al., 2014).

### Virus growth curve

Monolayer cultures of mouse NA cells were infected with RABV rHep-Flury and Hep-dG at a multiplicity of infection (MOI) of 0.01. The cells were cultured in a 37°C incubator, and then the supernatants were collected at 24, 48, 72, and 96 h post-infection (p.i.). Virus titers were determined by fluorescent-focus assay as previously described (Liu et al., 2014).

### Virus infection

NA cells were inoculated with RABV rHep-Flury and Hep-dG at MOI of 0.01, each experimental and control group was prepared in two biological replicates. The rate of virus infection was monitored by immunofluorescence, as previously described (Zandi et al., 2009). Briefly, after 24, 48, 72, and 96 h, cells infected with RABV rHep-Flury and Hep-dG were washed with PBS and fixed with 80% cold acetone at −20°C for 30 min. Afterwards, the cells were stained with FITC-labeled anti-rabies mAb (Fujirabio, USA) at 37°C for 2 h. Finally, infected cells were quantified by fluorescence microscopy.

### Cell viability assay

Cell viability was assessed with the cell counting kit-8 (CCK-8, Beyotime Biotech, China) according to the manufacturer's instructions. Briefly, NA cells were seeded into 96-well plates and incubated at 37°C. After 24 h pre-incubation, cells were treated with RABV rHep-Flury and Hep-dG at various MOI of 0.01, 0.1, and 1. Six replicate wells were used for the control and each test group. After 48 h post-infection, 10 μl of CCK-8 was added to each well, followed by additional incubation at 37°C for 2 h. Finally, absorbance at 450 nm was measured using a microplate reader.

### Cell apoptosis assessment

Cell apoptosis was quantified by using an Annexin V-FITC apoptosis kit (Vazyme Biotech, China) according to the manufacturer's instructions. Briefly, NA cells were seeded into 6-well plates and incubated at 37°C. At a density of 80%, cells were treated with RABV rHep-Flury and Hep-dG at MOI of 0.01. Then, cells were collected and incubated with 5 μL Annexin V and 5 μL PI for 10 min at 48 h post-infection. Afterwards, 500 μl of binding buffer was added to each tube, and the samples were analyzed using flow cytometry (BD FACS Aria, USA) within 1 h.

### Protein extraction and digestion

Protein extraction and digestion were performed according to the Filter-Aided Sample Prep (FASP) procedure as previously described (Wisniewski et al., 2009). After 48 h post-infection, NA cells from each experimental and control group (200 μg of total protein) were lysed with 30 μL SDT buffer (4% SDS, 100 mM DTT, 150 mM Tris-HCl, pH 8.0), and boiled for 15 min. The samples were diluted with 200 μL UA buffer (8 M Urea, 150 mM Tris-HCl pH 8.0) after equilibration to room temperature. The protein solution was concentrated by ultrafiltration through a 30 kD filter (Sartorius, German). Subsequently, 100 μl of 0.05 M iodoacetamide in UA buffer was added, and the mixture was incubated for 30 min in the dark. After centrifugation, the filters were washed thrice with 100 μl UA buffer and twice with 100 μl DS buffer (50 mM triethylammonium bicarbonate, pH 8.5). Finally, the protein suspensions were digested with 2 μg trypsin (Promega, Madison, WI) in 40 μl DS buffer overnight at 37°C. The resulting peptides were collected as a filtrate and the peptide content was determined by measuring the absorbance at 280 nm.

### iTRAQ labeling

The resulting peptide mixture was labeled with the iTRAQ Reagent-8plex Multiplex Kit (Applied Biosystems, Foster City, CA) according to the manufacturer's instructions. Briefly, iTRAQ reagents in 70 μl ethanol were added to the respective peptide mixture. Then, control samples (two biological replicates) were labeled with tags 113 and 116, respectively. In parallel, 2 biological replicate samples of rHep-Flury-infected cells were labeled with tags 114 and 117, respectively, while two biological replicate samples of Hep-dG -infected cells were labeled with tags 115 and 118, respectively. Finally, the labeled samples were incubated at room temperature for 1 h, and dried by vacuum centrifugation.

### Strong cationic exchange (SCX) fractionation

iTRAQ labeled peptides were fractionated by SCX chromatography using AKTA purifier 100 (GE Healthcare, UK) according to the manufacturer's instructions. Briefly, dried peptide mixtures were reconstituted and acidified with 2 ml buffer A (10 mM KH_2_PO_4_ in 25% of ACN, pH 2.7). The sample was loaded onto a Polysulfoethyl 4.6 × 100 mm column (5 μm, 200 Å, PolyLC Inc, Maryland, USA). Then, the peptides were eluted with a linear gradient of 0–10% buffer B (10 mM KH_2_PO_4_ in 25% of ACN, 500 mM KCl, pH 2.7) for 2 min, 10–20% buffer B for 25 min, 20–45% buffer B for 5 min, and 50–100% buffer B for 5 min at a flow rate of 1 ml/min. UV absorbance was monitored at 214 nm, while fractions were collected every 60 s. Ten sample pools containing 30 collected fractions were desalted on C18 Cartridges (Sigma, USA). Each fraction was dried in a vacuum centrifuge. Finally, samples were reconstituted in 40 μl of 0.1% (v/v) trifluoroacetic acid and stored at −80°C until LC-MS/MS analysis.

### Liquid chromatography (LC)—electrospray ionization (ESI) tandem MS (MS/MS) analysis by Q exactive

The assay was performed on a Q exactive mass spectrometer (Thermo Finnigan, San Jose, CA) coupled to Easy nLC (Thermo Fisher Scientific, USA). Briefly, 10 μl of each fraction were injected for nanoLC-MS/MS analysis. Then, 5 μg peptide mixtures were loaded on a C18 RP column (Thermo Scientific, 10 cm long, 75 μm inner diameter, 3 μm resin) and washed in buffer A (0.1% Formic acid). Elution of peptides was separated with a linear gradient of buffer B (80% acetonitrile and 0.1% Formic acid) at a flow rate of 250 nl/min, which was controlled by the IntelliFlow technology over 140 min. Then, a data-dependent top10 method was applied to select the most abundant precursor ions (mass range 300–1800 m/z). Determination of the target value was based on predictive Automatic Gain Control (pAGC), and dynamic exclusion duration was 60 s. Survey scans were acquired at a resolution of 70,000 at m/z 200 and resolution for HCD spectra was set to 17,500 at m/z 200. Normalized collision energy was 30% and the underfill ratio was defined as 0.1%. The instrument was run with the peptide recognition mode enabled (Fan et al., 2014).

### Protein identification, quantification, and bioinformatics

All raw files were searched using the MASCOT engine (Matrix Science, London, UK; version 2.2), and embedded into Proteome Discoverer 1.4 (Thermo Electron, San Jose, CA). Searches were against the uniprot-mouse database (3/8/2013, 73,952 sequences). The reversed sequences were used as decoy (Sandberg et al., 2012).

For protein identification, database search for each set was performed by Mascot 2.2 using the following search parameters: Peptide mass tolerance = 20 ppm, MS/MS tolerance = 0.1 Da, Enzyme = trypsin, Missed cleavage = 2. Fixed modifications: carbamidomethyl (C), iTRAQ 8-plex (K), iTRAQ 8-plex (N-term), variable modification: oxidation (M).

For protein quantitation, ratios were calculated and derived by Proteome Discover 1.4 using the following search parameters: Peptide false discovery rate ≤ 0.01 (Hoehenwarter et al., 2013), use only unique peptides, reject all quantification values if not all quantification channels are present. Final ratios were normalized by the median average protein quantification ratio for all eight labeled samples that served as sample REF (Yang et al., 2015). The correction method is based on the assumption that the expression of most proteins does not change. Subsequently, the meaningful cutoff for up-regulation and down-regulation of proteins was finalized using a population statistics applied to the biological replicates as proposed by Gan et al. (2007) and Ghosh et al. (2013).

Accession numbers of significantly altered proteins in Hep-dG-infected cells were imported into the Ingenuity Pathway Analysis software (IPA, www.ingenuity.com) for bioinformatics analysis and scientific findings curated from the literature including canonical pathways, diseases and biological functions, tox functions, networks, and upstream regulators, as described previously (Rajagopalan and Agarwal, 2005).

### Western blot analysis

Monolayer cultures of 8 × 10^6^ NA cells were infected with Hep-dG and rHep-Flury at a MOI of 0.01. At 48 h post-infection, cells were collected and lysed with RIPA and 1% PMSF (Beyotime Biotech, China). The concentrations of extracted proteins were measured by the Pierce BCA protein assay kit (Thermo scientific, USA) according to the manufacturer's instructions. A total of 40μg of protein were resolved by 12% SDS-PAGE electrophoresis and transferred onto a polyvinylidene difluoride (PVDF) membrane (Millipore, USA). The membranes were blocked by 5% skim milk powder in TBST for 1 h at room temperature. Afterwards, the membranes were incubated overnight at 4°C with primary antibodies raised in rabbits against ISG15, STAT1, p-STAT1(Y701), p-STAT3(Y705), and GAPDH (1:500 dilutions, Bioworld Technology, USA); STAT3, RIG-I, and LC3(1:1000 dilutions, Cell Signaling Technology, USA). Other primary antibodies included mouse anti-rabies virus G, N, P monoclonal antibodies (1:500 dilutions, Tongdian Biotechnology, China). After three washes with TBST, membranes were incubated with secondary goat anti-mouse IgG (H+L) or goat anti- rabbit IgG (H+L) conjugated to HRP (1:50,000 dilutions, Bioworld Technology, USA) at 37°C for 2 h. Finally, target proteins were detected by BeyoECL plus (Beyotime Biotech, China) according to the manufacturer's instructions and densitometry was assessed with the Image J software (NIH).

### Quantitative real time PCR (qRT-PCR)

NA cells were cultured and infected with Hep-dG and rHep-Flury in the same way as for proteomics analysis. At 48 h post-infection, cells were collected and harvested and total RNA was extracted for examination of IFN-β gene expression by qRT-PCR as previously described (Liu et al., 2012a). Briefly, Total RNA was extracted from RABV-infected cells with the TRIzol Reagent (LIFE, USA). First strand cDNA was generated from total RNA using the ReverTra Ace qPCR RT Master Mix with gDNA Remover (TOYOBO, JAPAN) following the manufacturer's protocol. The specific primers used in this study were: IFN-β Forward, 5′-ACCACAGCCCTCTCCATCAA -3′ and Reverse, 5′-TTGAAGTCCGCCCTGTAGGT-3′; GAPDH Forward, 5′-AGAG TGTTTCCTCGTCCCGT-3′ and Reverse, 5′-CTGTGCCGTTGAATTTGCCG-3′. mRNA abundance was assessed using THUNDERBIRD SYBR qPCR Mix (TOYOBO, JAPAN) and the iQ5 iCycler detection system (Bio-Rad) in three independent replicates. qRT-PCR conditions were as follows: 95°C for 1 min, followed by 40 cycles of 95°C for 15 s and 60°C for 35 s. The relative expression of mRNA was calculated by the traditional 2^−ΔΔct^ method (Livak and Schmittgen, 2001) after normalization to GAPDH.

## Results

### Viral growth of RABV rHep-flury and Hep-dG in NA cells

**Figure 1:**
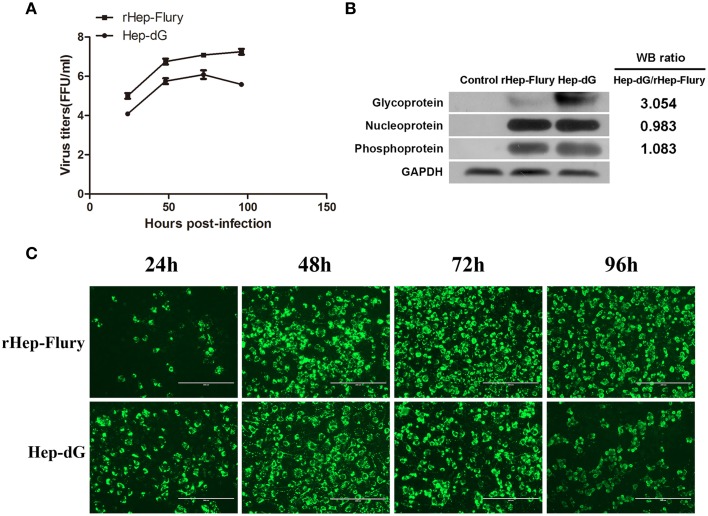
**Lower virus titers and higher G protein expression levels in Hep-dG-infected NA cells. (A)** The replication of rabies viruses rHep-Flury and Hep-dG in NA cells. NA cells were infected with rHep-Flury (■) and Hep-dG (•) at a MOI of 0.01. Supernatants were harvested at 24, 48, 72, and 96 h post-infection, and virus titers were determined by fluorescent-focus assay. Data were mean ± SEM. *n* = 3. **(B)** Rabies virus structural protein in NA cells were quantified by western blotting. Densitometry of the western blotting was analyzed with the Image J software (NIH). **(C)** Virus infection rate in NA Cells. NA cells were infected with rHep-Flury and Hep-dG at a MOI of 0.01. After 24, 48, 72, and 96 h of viral inoculation, FITC anti-rabies monoclonal globulin was used for staining. Images were taken at an original magnification of 200×.

The replication of RABV rHep-Flury and Hep-dG in NA cells was examined by single step growth curves. As shown in Figure 1A, the virus titers of the rHep-Flury were higher than those obtained for Hep-dG at each time point. Indeed, maximum virus titers of rHep-Flury and Hep-dG were 1 × 10^7.25^ and 1 × 10^6.10^ FFU/ml in NA cells, respectively. At 24, 48, 72, and 96 h post-infection, the titers of the rHep-Flury virus in NA cells were significantly higher than those of Hep-dG virus (*p* < 0.05, independent sample *t*-test using the Statistical Package for Social Sciences (SPSS), version 17.0).

The expression of various rabies virus structural proteins was also assessed in NA cells (Figure 1B) using western blotting. The results showed that the expression levels of RABV N and P proteins were nearly identical between rHep-Flury and Hep-dG-infected cells. However, the expression levels of RABV G protein in Hep-dG-infected cells were three times higher than that in rHep-Flury-infected cells.

Because rabies virus does not induce a typical cytopathic effect (CPE) in NA cells, viral infection rate was assessed by the detection of rabies virus antigen using immunofluorescence at 24, 48, 72, and 96 h p.i. Quantitative proteomic analysis was used to investigate the potential changes to the host cell proteome during rabies virus infection and replication, thus required a high proportion of RABV-infected NA cells (Fan et al., 2012). Direct immunofluorescence results showed that more than 80% NA cells inoculated with both rabies rHep-Flury and Hep-dG viruses were infected at 48 h p.i (Figure 1C). Since NA cell infection with Hep-dG resulted in high cell death at 72 h p.i (data not shown), the 48 h time point was chosen for proteomic analysis (Zandi et al., 2009).

### Cell viability

**Figure 2:**
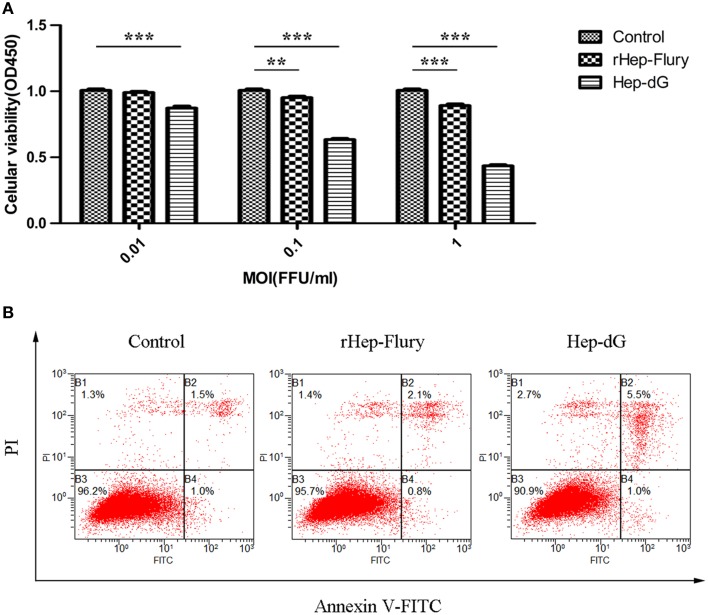
**Higher apoptosis rates in Hep-dG-infected NA cells. (A)** Effects of rabies viruses rHep-Flury and Hep-dG on cell viability. NA cells were infected with rHep-Flury and Hep-dG at various MOI of 0.01, 0.1, and 1. Cell viability was assessed with the cell counting kit-8. Absorbance at 450 nm was measured using a microplate reader. Data were mean ± SEM. *n* = 6. Differences (independent-samples *t*-Test) being ^**^p < 0.01, and ^***^p < 0.001. **(B)** Cell apoptosis induced by rabies viruses Hep-dG and rHep-Flury. NA cells were infected with rHep-Flury and Hep-dG at a MOI of 0.01. Cell apoptosis was quantified by using Annexin V-FITC apoptosis kit. Samples were analyzed by flow cytometry. The lower right part (Annexin V+/PI-) was considered as early stage of apoptotic cells and top right part (Annexin V-/PI+) was considered as late stage of apoptotic cells. The lower left part (Annexin V-/PI-) was considered as viable cells and the upper left part (Annexin V-/PI+) was considered as necrotic cells.

Cell viability was assessed using the CCK-8 assay. NA cells infected with either rabies virus showed a dose-dependent decrease in cell viability (Figure 2A). However, the viability of Hep-dG-infected cells was extremely and significantly reduced compared with the rHep-Flury group at various MOI of 0.01, 0.1, and 1 (*P* < 0.001, independent sample *t*-test using SPSS). Interestingly, at a very low MOI of 0.01, Hep-dG-infected cells showed a significant decrease in the viability, while rHep-Flury did not cause toxicity in these conditions. Since infection of Hep-dG induces significant cell death at MOI of 0. 1 or 1, MOI = 0.01 was chosen in the iTRAQ experiment.

### Cell apoptosis

To analyze cell apoptosis induced by rHep-Flury and Hep-dG, the NA cells infected with individual rabies virus were treated with Annexin V/PI. Hep-dG induced 6.5% NA cell apoptosis; however, rHep-Flury only induced 2.9% NA cell apoptosis (Figure 2B). These results indicated that the Hep-dG virus induced a higher apoptosis rate than rHep-Flury in NA cells (*p* < 0.01, Chi-square test using SPSS).

### Protein identification and quantification by iTRAQ

No previous study has used iTRAQ combined with LC-MS/MS approach to identify and quantify the differential protein expression profiles of NA cells infected with recombinant RABV. Interestingly, Hep-dG-infected cells yielded more distinct identified proteins than rHep-Flury-infected cells. In total, 24,869 peptides and 3072 proteins were detected, respectively. These identified proteins were then filtered using a population statistic to obtain the list of significantly altered proteins (Gan et al., 2007; Ghosh et al., 2013). Applying this method to our data sets, we observed about 19% variation for two rHep-Flury-infected groups (Figures S1A,B) and 20% variation for two Hep-dG-infected groups (Figures S1C,D) corresponding to 88% coverage of data. Based on this, the cutoff was fixed at 1.19-fold (19% variation) corresponding to the ITRAQ ratio of >1.19 for up-regulation and <0.84 (1/1.19) for down-regulation for two rHep-Flury-infected groups. Similarly for two Hep-dG-infected groups, the up-regulation cutoff was fixed as >1.20, and the down-regulation cutoff was fixed as <0.83. These cutoff thresholds were then applied to the average of four biological replicates to minimize false positive for determination of up- or down-regulated proteins. Finally, proteins that qualified these cutoff values in respective data sets were chosen as significantly altered ones (Ghosh et al., 2013).

**Figure 3:**
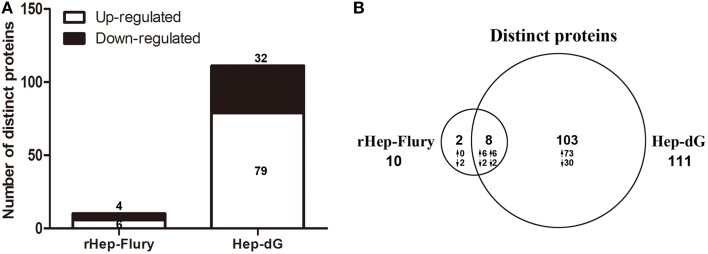
**An overview of differentially expressed proteins in rabies viruses rHep-Flury and Hep-dG infection group. (A)** Among the significantly altered proteins, 10 exhibited a differential expression pattern in rHep-Flury-infected cells compared to mock-infected cells. Of these, 4 proteins were highly down-regulated and 6 significantly up-regulated. Meanwhile, 111 proteins were differentially expressed in Hep-dG-infected cells compared to mock- infected cells. Of these, 32 proteins were highly down-regulated and 79 significantly up-regulated. **(B)** Venn diagrams depict the overlap of distinct proteins identification between rHep-Flury and Hep-dG-infected group. The number of proteins that have higher (↑) or lower (↓) fold-change values in each comparison are also shown.

Among the significantly altered proteins, 10 exhibited a differential expression pattern in rHep-Flury-infected cells compared to mock-infected cells (Supporting Information Table S1), of which 4 down-regulated and 6 up-regulated (Figure 3A). Meanwhile, 111 proteins were differentially expressed in Hep-dG-infected cells compared to mock- infected cells (Supporting Information Table S2), 32 being down-regulated and 79 up-regulated (Figure 3A). As shown in the Venn diagram, eight/113 differentially expressed proteins were common in both infection groups (Figure 3B).

### Ingenuity pathway analysis

Since Hep-dG-infected cells yielded more distinct identified proteins than rHep-Flury-infected cells, Ingenuity Pathway Analysis (IPA) was performed to gain further insights into differentially expressed proteins in Hep-dG-infected cells. The results, including main canonical pathways, diseases and biological functions, tox functions, networks, and upstream regulators, are described below.

**Figure 4:**
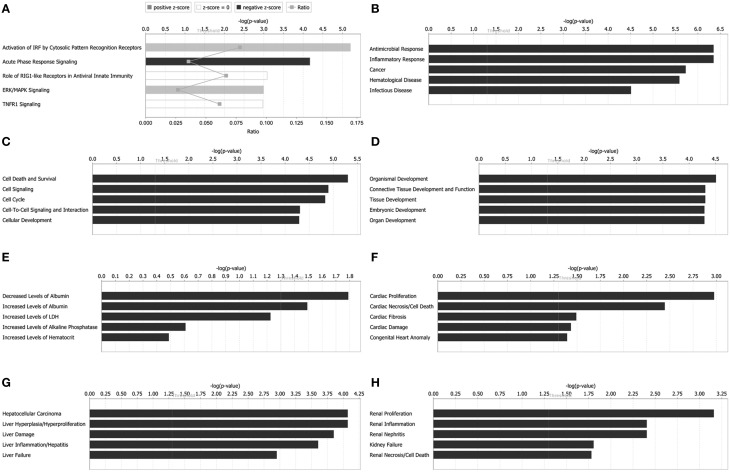
**Functional characterizations of differentially expressed proteins in rabies virus Hep-dG infection group. (A)** Top canonical pathways. **(B)** Diseases and disorders. **(C)** Molecular and cellular functions. **(D)** Physiological system development and functions. **(E)** Clinical chemistry and hematology. **(F)** Cardiotoxicity. **(G)** Hepatotoxicity. **(H)** Nephrotoxicity.

Canonical pathways analysis can predict the significantly regulated pathways base on the submitted information of altered proteins. Top canonical pathways in Hep-dG-infected cells comprised activation of IRF by cytosolic pattern recognition receptors (5 out of a possible 64 molecules, *P* = 6.31 × 10^−6^), acute phase response signaling (6 out of a possible 169 molecules, *P* = 6.68 × 10^−5^), role of RIG1-like receptors in antiviral innate immunity (3 out of a possible 45 molecules, *P* = 8.21 × 10^−4^), ERK/MAPK signaling (5 out of a possible 187 molecules, *P* = 1.01 × 10^−3^), and TNFR1 signaling (3 out of a possible 49 molecules, *P* = 1.05 × 10^−3^) (Figure 4A).

Downstream effects analysis can identify whether significant downstream biological processes are increased or decreased based on protein expression results, it includes diseases and biological functions and tox functions analysis.

Diseases and biological functions, including diseases and disorders, molecular and cellular functions, and physiological system development, were identified at *p* < 0.05. In Hep-dG-infected cells, top diseases and disorders included antimicrobial response, inflammatory response, cancer, hematological disease, and infectious disease (Figure 4B). Top molecular and cellular functions comprised cell death and survival, cell signaling, cell cycle, cell-to-cell signaling and interaction, and cellular development (Figure 4C). Top physiological system development and function included organismal development, connective tissue development and function, tissue development, embryonic development, and organ development (Figure 4D).

Tox functions, including clinical chemistry and hematology, cardiotoxicity, hepatotoxicity, and nephrotoxicity, were identified at *p* < 0.05. In Hep-dG-infected cells, top clinical chemistry and hematology included decreased levels of albumin, increased levels of albumin, increased levels of LDH, increased levels of alkaline phosphatase, and increased levels of hematocrit (Figure 4E). Top cardiotoxicity included cardiac proliferation, cardiac necrosis/cell death, cardiac fibrosis, cardiac damage, and congenital heart anomaly (Figure 4F). Top hepatotoxicity included hepatocellular carcinoma, liver hyperplasia/hyperproliferation, liver damage, liver inflammation/hepatitis, and liver failure (Figure 4G). Top nephrotoxicity included renal proliferation, renal inflammation, renal nephritis, kidney failure, and renal necrosis/cell death (Figure 4H).

**Figure 5:**
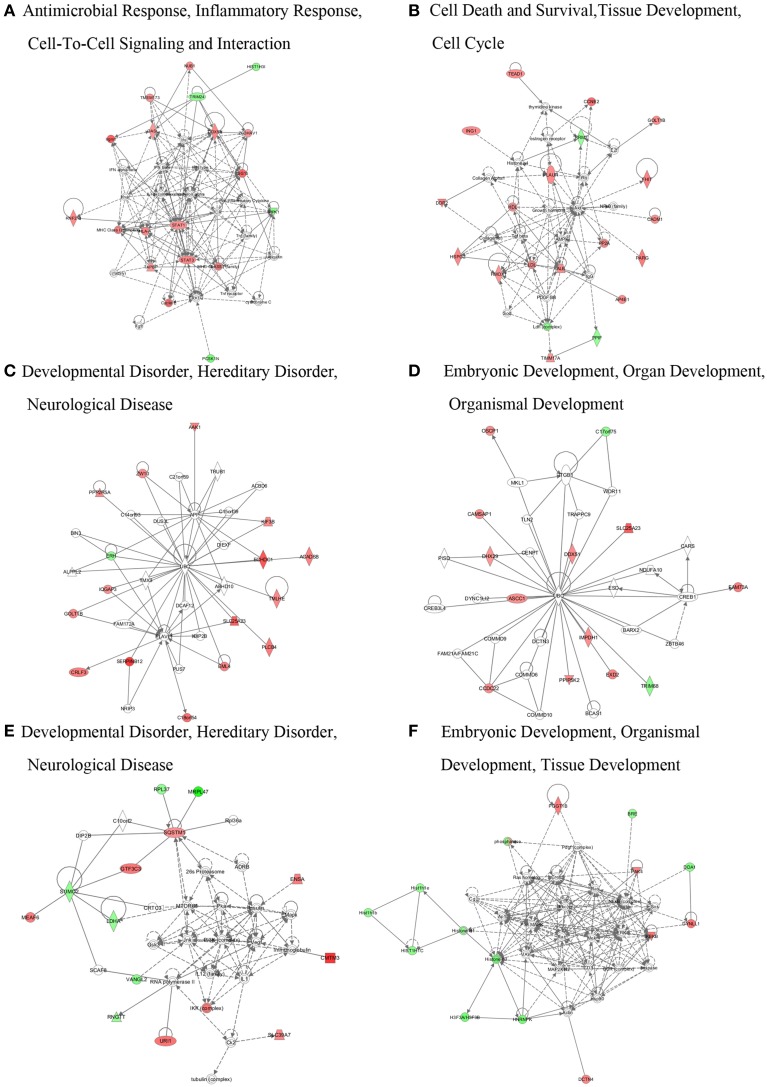
**Network analyses of differentially expressed proteins in rabies virus HEP-dG infection group. (A)** Antimicrobial response, inflammatory response, cell-to-cell signaling and interaction. **(B)** Cell death and survival, tissue development, cell cycle. **(C)** Developmental disorder, hereditary disorder, neurological disease. **(D)** Embryonic development, organ development, organismal development. **(E)** Developmental disorder, hereditary disorder, neurological disease. **(F)** Embryonic development, organismal development, tissue development. Red, significantly up-regulated proteins; green, significantly down-regulated proteins; and white, proteins known to be in the network but that were not identified in our study. Lines connecting the molecules indicate molecular relationships. Dashed lines indicate indirect interactions, and solid lines indicate direct interactions. The arrow styles indicate specific molecular relationships and the directionality of the interaction. More information is available in Table S3 in the Supporting Information.

Network analysis can build and explore protein-protein interaction network involved in our proteomics data. All significantly altered proteins were subjected to network analysis. Distinct proteins in Hep-dG-infected cells were mapped to 6 specific functional networks (Figure 5, Supporting Information Table S3). Interestingly, 16 proteins in top one network, which corresponded to antimicrobial response, inflammatory response, cell-to-cell signaling and interaction, were significantly altered in Hep-dG-infected cells, but not in the rHep-Flury infection group (Figure 5A). Moreover, most of these proteins were located upstream or downstream of type I interferon (Table 1). These data suggested that infection with rabies virus Hep-dG induces a more pronounced immune response compared with that with rHep-Flury.

**Table 1:** **Interferon stimulated proteins that were only significantly altered in Hep-dG infected NA cells^a^**.

**Protein name**	**Gene symbol**	**Accession number**	**Unique peptides**	**ITRAQ Ratios (Mean ± S.E.)**	**Expression pattern**
2′-5′-oligoadenylate synthase-like protein 1	Oasl1	Q8VI94	2	1.212 ± 0.011	↑
Zinc finger CCCH-type antiviral protein 1	Zc3hav1	Q3UPF5	8	1.215 ± 0.013	↑
Tapasin	Tapbp	G3UZZ2	2	1.240 ± 0.015	↑
Putative uncharacterized protein	Hmox1	Q3U5H8	6	1.250 ± 0.017	↑
Stimulator of interferon genes protein	Tmem173	Q3TBT3	1	1.259 ± 0.027	↑
Stat1 protein	Stat1	Q99K94	3	1.283 ± 0.020	↑
Sequestosome-1	Sqstm1	Q64337	8	1.301 ± 0.017	↑
NEDD8 ultimate buster 1	Nub1	P54729	4	1.318 ± 0.007	↑
MHC class I antigen	H2-K	K7WBQ4	3	1.375 ± 0.026	↑
Ddx58 protein	Ddx58	A1L0V6	2	1.378 ± 0.034	↑
E3 ubiquitin-protein ligase RNF213	Rnf213	E9Q555	16	1.391 ± 0.044	↑
Signal transducer and activator of transcription 3	Stat3	Q6GU23	2	1.415 ± 0.017	↑
mRNA	H2-D1	Q61892	1	1.454 ± 0.046	↑
Discoidin domain-containing receptor 2	Ddr2	Q62371	2	1.490 ± 0.053	↑
MHC class I antigen	H2-D	K7W4D1	2	1.551 ± 0.052	↑
Ubiquitin-like protein ISG15	Isg15	Q64339	2	1.807 ± 0.114	↑
Immunity-related GTPase family M protein 1	Irgm1	Q5NCB5	3	1.861 ± 0.054	↑

a *Accession number provided from the uniprot mouse database (3/8/2013, 73,952 sequences). Detailed information can be found in Table S2*.

**Figure 6:**
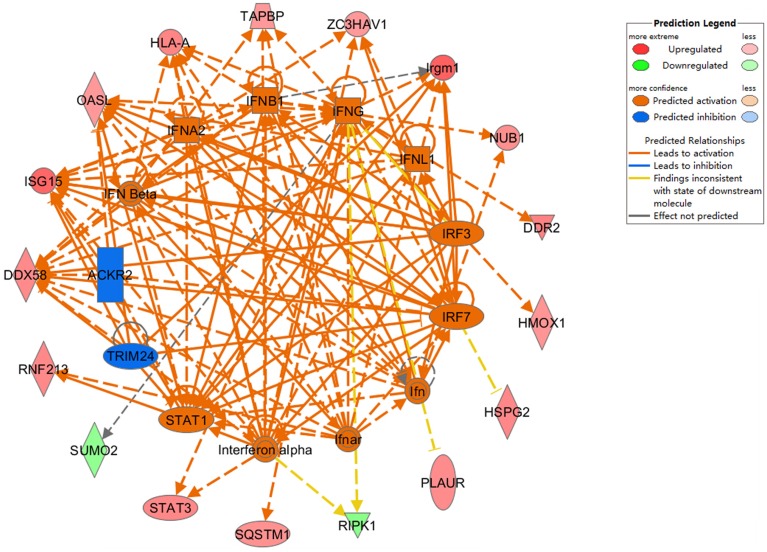
**Upstream analyses of differentially expressed proteins in rabies virus Hep-dG infection group**. Red, significantly up-regulated proteins; Green, significantly down-regulated proteins; Orange, predicted activation; Blue, predicted inhibition. Lines connecting the molecules indicate molecular relationships. Orange line, lead to activation; Blue line, lead to inhibition; Yellow line, findings inconsistent with state of downstream molecule; Gray line, effect not predicted. More information is available in Table S4 in the Supporting Information.

To further assess protein alterations detected by iTRAQ, the potential upstream regulators were analyzed. Upstream regulator analysis can predict upstream molecules, which may cause changes in the observed protein expression. The identified upstream regulators were predicted to be activated or inhibited based on activation z-score. Interestingly, based on expression change of multiple interferon-stimulated proteins, 11 upstream regulators were predicted to be activated in Hep-dG infected cells, including IFN Beta, IFNA2, IFNB1, IFNG, IFNL1, IRF3, IRF7, Ifn, Ifnar, Interferon alpha, STAT1, while 2 upstream regulators were predicted to be inhibited, including TRIM24 and ACKR2 (Figure 6, Supporting Information Table S4). In addition, two MHC class I molecules (H2-K and H2-D), which cannot be annotated by IPA software, were significantly up-regulated only in the Hep-dG-infected cells. These results suggested that interferon signaling pathways may be strongly activated in the Hep-dG infection group, but not in rHep-Flury-infected cells.

### Validation of various protein level alterations by western blotting

**Figure 7:**
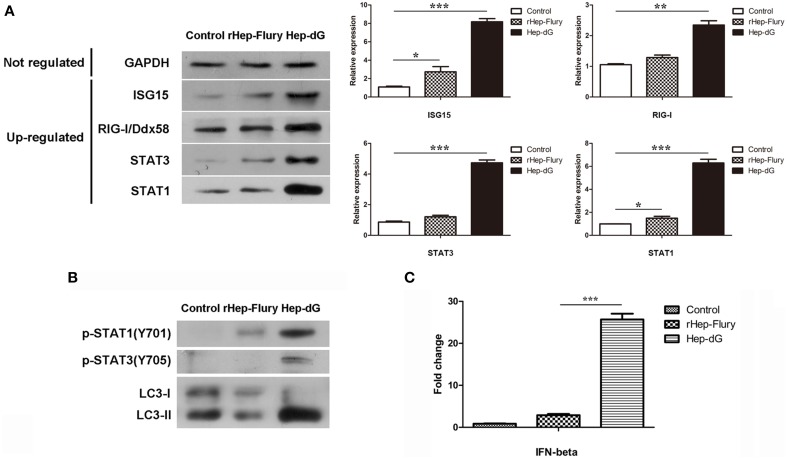
**Validation of selected data by western blot and qRT-PCR. (A)** Representative proteins in infected and mock-infected NA cells were quantified by western blotting. Densitometry of the western blotting was analyzed with the Image J software (NIH). Values are expressed relative to GADPH. Data are mean ± SEM. *n* = 3. Differences (independent-samples *t*-test) being ^*^p < 0.05, ^**^p < 0.01, and ^***^p < 0.001. **(B)** p-STAT1 (T701), p-STAT3 (T705) and autophagy-related markers LC3 in NA cells were quantified by western blotting. **(C)** IFN gene expression in rabies viruses rHep-Flury and Hep-dG-infected NA cells. GAPDH was used as the internal loading control. Data are mean ± SEM. *n* = 3. Differences (independent-samples *t*-test) being ^***^p < 0.001.

To confirm the differentially expressed proteins identified by the iTRAQ method, several up-regulated and unaltered proteins between infected and mock-infected cells were quantified by western blotting. As shown in Figure 7A, the levels of seven representative proteins, including GAPDH, ISG15, RIG-I/Ddx58, STAT1, and STAT3, were consistent with iTRAQ results.

To further confirm the prediction of interferon signaling activation in NA cells, p-STAT1(T701) and p-STAT3(T705) expression levels were assessed by western blot, since crucial tyrosine residues for STAT1 and STAT3 activation are located at positions 701 and 705, respectively (Regis et al., 2008) (Figure 7B). Compared with the rHep-Flury infection group, the phosphorylation level of STAT1 in Hep-dG-infected cells was significantly increased. Meanwhile, STAT3 was phosphorylated only in Hep-dG-infected cells. Since up-regulation of many autophagy-associated proteins was observed, the expression of the autophagy-related marker LC3 was also assessed by western blot (Figure 7B). The results showed that rabies virus Hep-dG infection significantly increased LC3-I/LC3-II conversion in NA cells, indicating that over-expression of RABV G protein may induce NA cell autophagy.

### Determination of IFNs by quantitative real time PCR

Although significant changes were identified in the expression of stimulator of interferon genes protein (STING, also called TMEM173) and multiple interferon-stimulated proteins, we were unable to detected IFNs by LC-MS/MS. In order to assess whether expression of IFNs was increased by rabies virus Hep-dG infection in NA cells, we examined the expression of IFN-β by qRT-PCR, a method commonly used in equantifying IFN expression in virus infected cells (Liu et al., 2012a). Interestingly, IFN-β mRNA levels in Hep-dG-infected cells were approximately eight-fold higher than those obtained for the rHep-Flury infection group (*P* < 0.001, independent-samples *t*-test using SPSS), as shown in Figure 7C. Combined with the proteomic data showing up-regulated expression of multiple interferon-stimulated proteins in the Hep-dG infection group, the increased IFN gene expression suggested that rabies virus Hep-dG infection induced type I IFN protein expression in NA cells.

## Discussion

Studies have shown that G protein is not only the major contributor to rabies virus pathogenicity but also the main antigen responsible for the induction of protective immunity (Flamand et al., 1993; Zhang et al., 2013a). The recombinant rabies viruses SAD B19 (Faber et al., 2002) and RC-HL (Hosokawa-Muto et al., 2006) carrying two copies of the G gene has been reported previously. Similarly, our lab has constructed a recombinant RABV Hep-dG strain, which encodes two identical copies of the G-gene and improves G protein expression in BHK-21 cells (Liu et al., 2014); this was obtained also for NA cells (Figure 1B). Interestingly, rabies virus Hep-dG induced higher apoptosis rates (Figure 2) and lower virus titers than rHep-Flury in NA cells (Figure 1A), while higher NA cell infection rates were obtained after inoculation with Hep-dG compared with rHep-Flury at 24 h post-infection (Figure 1C). It is likely that increased glycoprotein expression lead to more efficient receptor-mediated uptake in the early stages of infection (Liu et al., 2014), while cell apoptosis or other host anti-virus mechanisms could lead to decreased virus titers.

Proteomics is a useful tool for comprehensive and quantitative analysis of cellular protein expression. It has been widely used in many research fields. We used the iTRAQ technology and expanded this work to explore the link between the expression level of RABV glycoprotein and immunogenicity of the viral infection. Surprisingly, compared with rHep-Flury-infected cells, Hep-dG-infected cells showed a significant increase on distinct proteins (Figure 3). A total of 111 differentially expressed proteins were identified in Hep-dG-infected cells. However, only 10 differentially expressed proteins were identified in rHep-Flury-infected cells. In addition to the common set of 8 proteins identified in both RABV infection groups, 103 proteins were significantly altered only in the Hep-dG infection group (Figure 3B).

Network and upstream analysis using the IPA software predicted that the interferon signaling may be activated in Hep-dG-infected cells, but not in the rHep-Flury infection group (Figures 5, 6). The principal response of host cells to viral infection is the induction of type I interferons, which can be produced in many cell types and establish an anti-viral state through the induction of IFN-stimulated genes (ISGs) (Liu et al., 2012b). It has been reported that rabies virus infection is recognized by the cellular sensor protein RIG-I (Hornung et al., 2006). Interaction between RIG-I and the mitochondrial adaptor protein VISA results in activation of TBK-1 and phosphorylation of IRF-3; then, phosphorylated IRF-3 and NF-κB work synergistically to induce the production of type I interferons, and activate the JAK/STAT intracellular signaling pathway (Akira et al., 2006; Honda and Taniguchi, 2006; Katze et al., 2008). We have demonstrated herein that STING and a lot of interferon-stimulated proteins were significantly up-regulated only in the Hep-dG infection group (Table 1). Together with the qRT-PCR data, the major findings of the proteomic analysis in our study was that over-expression of RABV G protein could induce more type I interferon protein expression in NA cells, and activation of interferon signal and up-regulation of multiple interferon-stimulated proteins, which could contribute to innate and adaptive immunity during virus infection.

JAK-STAT signaling is the main signaling pathway activated by type I (IFN-α and IFN-β) and type II (IFN-γ) interferons. Studies have shown that STAT1 plays an important role in inflammation, growth arrest, and apoptosis induction; conversely, STAT3 promotes cell-cycle progression and cellular transformation and prevents apoptosis (Bromberg et al., 1999). Interestingly, our data showed up-regulation of both STAT1 and STAT3 in the Hep-dG infection group, but not in rHep-Flury-infected cells. Moreover, compared to the rHep-Flury infection group, the phosphorylation level of STAT1 in Hep-dG-infected cells was significantly increased. Meanwhile, STAT3 was phosphorylated only in the Hep-dG infection group. These data further demonstrated that over-expression of G protein can promote IFN production. Nevertheless, activation of STAT3 may be negative feedback to STAT1-dependent inflammatory gene activation as previously described (Ho and Ivashkiv, 2006).

MHC molecules play essential roles in the initiation (MHC class II) or effector phase (MHC class I) of the immune response by presenting peptides at the cell surface to CD8+ and CD4+ T cells, respectively (Vyas et al., 2008). MHC class I molecules are expressed by all nucleated cells, while MHC class II molecules are primarily expressed by professional APCs (Neefjes et al., 2011). Previous research has shown that class-I MHC molecules are expressed on neurons in a type I IFN-dependent fashion, after TMEV infection (Njenga et al., 1997). Similar to the above studies, we found that three of MHC class I molecules (H2-K, H2-D1, and H2-D) and tapasin, an ER chaperone that controls MHC class I assembly with peptides (Grandea and Van Kaer, 2001), were significantly up-regulated only in the Hep-dG infection group. These data indicated that over-expression of RABV G protein leads to increased expression of MHC class I molecules, which would facilitate host cell recognition and lysis RABV by -specific CD8+ T cells and induce antigen-specific T cell immune response.

Four interferon-stimulated proteins with antiviral function were up-regulated only in the Hep-dG infection group, including RIG-I, ISG15, Zc3hav1, and Irgm1. Up-regulation of RIG-I (also called Ddx58) in Hep-dG-infected cells was consistent with previous reports that rabies virus infection is recognized by RIG-I, which initiate antiviral responses by producing types I and III IFNs (Hornung et al., 2006; Onoguchi et al., 2011). ISG15 is one of the most strongly induced ISG proteins that can be covalently attached to both host and viral proteins, functioning by either disrupting the activity of a target protein or by altering its localization within the cell; therefore, ISG15 plays an important role in mammalian antiviral immunity (Skaug and Chen, 2010). Zc3hav1 (also called ZAP) acts through direct binding to viral RNA and recruits the processing exosome, eventually leading to viral RNA degradation (Guo et al., 2007). It is a key regulator of RIG-I-mediated induction of type I IFN and antiviral defenses (Hayakawa et al., 2011). Irgm1 (also called LRG47) is known to regulate host resistance to intracellular pathogens through multiple mechanisms, which includes promotion of autophagy-dependent killing of pathogens (Gutierrez et al., 2004). Up-regulation of these antiviral proteins was in line with virus growth curve (Figure 1A). The titers of the Hep-dG virus in NA cells were significantly lower than those of rHep-Flury virus. However, the titers of the Hep-dG virus in BHK-21 cells were higher than those of rHep-Flury virus (Liu et al., 2014). It indicates that, in addition to the over-expression of RABV G protein, the longer genome of Hep-dG may result in the inhibition of viral replication via IFN-dependent mechanism (Faber et al., 2002), but do not reduce its replication efficiency.

Apoptosis and autophagy are two distinct self-destructive processes; both influence the normal clearance of dying cells and immune recognition of dead cell antigens. Loss and gain of either autophagy or apoptosis influence numerous pathological processes, and these phenomena affect each other (Maiuri et al., 2007). Among the interferon-stimulated proteins, two apoptosis-associated molecules (including NUB1 and STAT1) and two autophagy-associated proteins (including SQSTM1 and Irgm1) were significantly up-regulated only in the Hep-dG infection group. NUB1 mediates anti-proliferative actions and apoptosis through cell-cycle regulation (Hosono et al., 2010). STAT1, specifically the C-terminus, has been shown to be involved in promoting apoptotic cell death (Janjua et al., 2002). Up-regulation of NUB1 and STAT1 protein was in line with the cell viability findings (Figure 2A) and FITC/PI data (Figure 2B), further demonstrating that over-expression of RABV G protein results in enhancement of apoptosis, in agreement with previous studies (Faber et al., 2002; Hosokawa-Muto et al., 2006; Liu et al., 2014). Among the autophagy-associated proteins, SQSTM1 (p62) serves as autophagy receptor for selective autophagic clearance of protein aggregates and organelles (Komatsu et al., 2012). Irgm1, mentioned for its antiviral function, has been shown to induce autophagy by acting on mitochondria (Singh et al., 2010). These results indicate that over-expression of RABV G protein may induce autophagy in NA cells, which was confirmed by western blot (Figure 7B). However, further studies should be carried out to explain the relationship between RABV G protein and autophagy.

## Conclusions

In summary, the proteomic changes in RABV-infected NA cells were characterized using ITRAQ coupled to LC-MS/MS. This proteome study has revealed that cellular factors corporately regulate the replication of RABV and host response to infection. Interestingly, the proteomic data show up-regulated expression of multiple interferon-stimulated proteins only in Hep-dG-infected cells, and the increased IFN gene expression together proved that Hep-dG induced more interferons than rHep-Flury in NA cells. Together, this study for the first time demonstrates a positive correlation between the amount of RABV glycoprotein expression and the increasing of type I interferon from the point of proteomics analysis. Furthermore, the up-regulation of multiple interferon-stimulated proteins suggested that over-expression of glycoprotein in the attenuated RABV strain can promote the antiviral response, MHC-I mediated antigen-specific T cell immune response, apoptosis, and autophagy in an IFN-dependent manner. These findings will be helpful for better understanding of molecular mechanisms of immune response during the RABV infection. What's more, these results again certified that the rabies virus Hep-dG strain could be excellent candidate to RABV vaccine.

### Conflict of interest statement

The authors declare that the research was conducted in the absence of any commercial or financial relationships that could be construed as a potential conflict of interest.
