# Supplementary material for: iTRAQ protein profile analysis of neuroblastoma (NA) cells infected with the rabies viruses rHep-Flury and Hep-dG
Source: Front Microbiol. 2015 Jul 7;6:691. doi: 10.3389/fmicb.2015.00691 (PMC4493837; doi:10.3389/fmicb.2015.00691)
Supplement: Supplementary file 5 [file Image1.PDF]

Figure S1：Determination of experimental variation using all identified proteins common in both biological replicates. Two biological replicates for each sample were used for labeling however analysis of the data set was performed by cross comparison between all four replications (replication1, 114:113 and 115:113; replication2, 114:116 and 115:116; replication3, 117:113 and 118:113; replication4, 117:116 and 118:116). Briefly, the variation between the averages of four replicates (sample versus control ratios) and one was calculated and converted to the corresponding percentage variation. These percentage variations were then plotted against the cumulative percentage coverage and the variation against 88% coverage was taken into account to determine the fold cutoff considering the population outside 88% as significantly altered (Gan et al., 2007; Ghosh et al., 2013). The horizontal axis represents % variation of ITRAQ ratios of same protein from different biological replicate samples. The primary vertical axis represents the corresponding number of proteins (bar) having different % variation. The secondary vertical axis represents the cumulative % of the counted proteins (line). Variation against 88% coverage of population was considered for selecting cutoff. **(A)** corresponds to the first rHep-Flury-infected group (replication1 114:113 and replication4 117:116). **(B)** corresponds to the second rHep-Flury-infected group (replication2 114:116 and replication3 117:113). **(C)** corresponds to the first Hep-dG-infected group (replication1 115:113 and replication4 118:116). **(D)** corresponds to the second Hep-dG-infected group (replication2 115:116 and replication3 118:113).

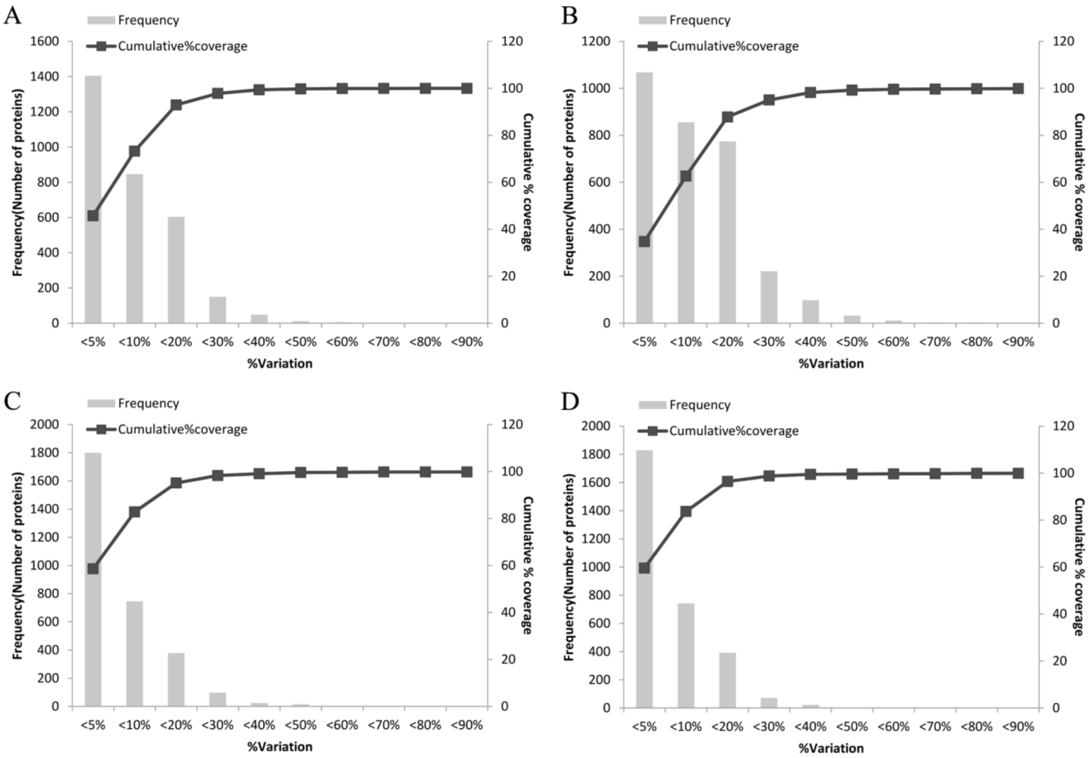

References:

Gan, C.S., Chong, P.K., Pham, T.K., and Wright, P.C. (2007). Technical, experimental, and biological variations in isobaric tags for relative and absolute

quantitation (iTRAQ). *J. Proteome Res.* 6, 821-827. doi: 10.1021/pr060474i.
Ghosh, D., Li, Z., Tan, X.F., Lim, T.K., Mao, Y., and Lin, Q. (2013). "iTRAQ Based Quantitative Proteomics Approach Validated the Role of Calcyclin Binding Protein (CacyBP) in Promoting Colorectal Cancer Metastasis", in.).
